# Evaluating Range of Motion of Two Prominent Neck Support Devices for Daily Activities

**DOI:** 10.1109/TNSRE.2025.3647266

**Published:** 2026

**Authors:** Kimia Khoshnami, Edoardo Battaglia, Mark Bromberg, Haohan Zhang

**Affiliations:** Department of Mechanical Engineering, University of Utah, Salt Lake City, UT 84112 USA; Department of Mechanical Engineering and the Robotics Center, University of Utah, Salt Lake City, UT 84112 USA; Department of Neurology, University of Utah, Salt Lake City, UT 84112 USA; Department of Mechanical Engineering and the Robotics Center, University of Utah, Salt Lake City, UT 84112 USA

**Keywords:** Powered neck exoskeleton, dropped head syndrome, physical human–robot interaction, amyotrophic lateral sclerosis

## Abstract

Neck muscle weakness causes the inability to raise and move the head, leading to fatigue, neck pain, and a “head-on-chest” posture (dropped head syndrome) in severe cases, which significantly affects quality of life. Static neck collars are the current standard of care. However, these collars are passive, which cannot restore the head-neck movement necessary for daily tasks. Emerging robotic devices like powered neck exoskeletons were developed to enable head-neck movements. Previous laboratory tests showed improved patients’ ability to follow prescribed trajectories; however, the ability to assist with daily tasks of such a robotic device remains unknown. In this paper, the functional range of motion allowed by a state-of-the-art powered neck exoskeleton was compared to a clinic-standard static neck collar in healthy adults performing simulated daily tasks wearing these devices. Results showed a greater head range of motion and consequently less compensatory torso movements while wearing the neck exoskeleton in its transparent mode. Participants rated the neck exoskeleton more favorably than the static collar in terms of comfort and ability to perform the tasks. Results also revealed the range of motion limits of the current neck exoskeleton for these daily tasks. These results provided justifications for using neck exoskeletons to restore daily functions and offered critical insights into future refinement of this technology to enable head range of motion for critical daily activities.

## I. Introduction

DROPPED head syndrome (DHS) is characterized by a person’s inability to move and raise their head. DHS results from neck muscle weakness, which can arise from diverse causes, including central/peripheral neurological pathology (e.g., amyotrophic lateral sclerosis, Parkinson’s disease) and autoimmune conditions (e.g., polymyositis) [1]. People with DHS cannot maintain an upright head posture for an extended period of time due to fatigue, and in severe cases, this condition results in a “head-on-chest” posture due to complete neck muscle weakness [2]. DHS causes pain and spinal deformity, as well as difficulty with respiratory functions, ambulation, and social interactions, which severely impacts patients’ physical and emotional well-being and overall quality of life [3]. A common treatment is using a static neck collar to hold the head in an upright position [4]. However, available collars are uncomfortable because they do not allow voluntary movements for activities of daily living [5], [6]. Additionally, these collars support the head at the chin and clavicle and often apply pressure around the neck, which interferes with speaking, swallowing, and breathing functions and causes further discomfort. Because of these limitations, few patients use their collars at home; they would rather leave their condition untreated, which worsens their quality of life [6], [7], [8].

There have been attempts to improve the comfort of static neck collars.

A prominent example is the HeadUp collar [9]. This device supports the head upright while adding small compliance using multiple flexible beams attached around the neck. The idea was that the flexible beams can act like springs such that a user can self-initiate small head movements around the upright posture, thereby improving comfort.

Two studies have been published to evaluate this collar in patients with DHS. The first study [9] gathered patient’s qualitative feedback using the HeadUp collar in their daily activities, as compared to other neck collars they have used. The second study [10] focused on a larger group of people to evaluate the patient-rated acceptability of the HeadUp collar. Results of both studies highlighted how an appropriately made neck collar can be helpful for patients experiencing DHS; however, they also revealed that the HeadUp collar may still be restrictive for important daily tasks from the patients’ perspective [9], [10].

To enable head-neck movements, powered neck exoskeleton devices have been developed [11], [12], [13], [14], [15]. These wearable devices use powers from external actuators (e.g., electric motors) to compensate for the neck muscle weakness and assist with the head-neck movements.

Despite the promise of such powered neck devices, few have been evaluated in patients with DHS. In fact, to the best of our knowledge, the only powered neck exoskeleton that has been evaluated in patients with DHS was previously developed by the senior author of this paper [16], [17], [18]. This neck exoskeleton used a parallel linkage mechanism to allow for the three rotations of the head relative to the trunk [19]. A structurally refined version of this exoskeleton has recently been built in our laboratory at the University of Utah (Utah Exo [20]). We have performed a study in six patients with amyotrophic lateral sclerosis where the Utah Exo was shown to allow for better kinematic performance (i.e., lower error) during a laboratory tracking task (e.g., following head trajectories) with lower muscular effort, as compared to using the previous design and to without relying on any powered assistance from the device [18].

While being assistive during specific laboratory tasks in patients with DHS, what is missing is the extent to which this neck exoskeleton can assist with activities of daily living, as compared to available static neck collars.

As shown in the literature [5], [21], head-neck movements are involved in many essential activities of daily living, such as looking for traffic before crossing a street and picking up an object from the floor. In addition to the involvement of the head-neck, other spinal segments of the body (i.e., thoracic and lumbar segments) are also involved in these real-world scenarios. As the mobility of the head is constrained by a neck support device, compensatory movements are likely to occur in these spinal segments [5].

As the first step towards our goal of evaluating the powered neck exoskeleton in patients with DHS for real-world tasks, in this paper, we focus on quantifying the effects of the structural constraints of the neck exoskeleton on head and torso range of motion (ROM) during simulated activities of daily living in a laboratory environment. To this end, we measured the ROM of the head and torso in healthy participants while wearing the exoskeleton in 8 simulated activities of daily living, selected based on literature [5], [21], and compared them with those using the HeadUp collar and those without any devices during the same tasks. To remove the potential confounding effects of the robot controllers on the measured ROM of the head and torso in healthy participants, the neck exoskeleton was operated in its “transparent” mode in which the exoskeleton does not apply noticeable forces on the head [22].

Here, our main contribution is quantifying, for the first time, the effects of the structure of the neck exoskeleton on the ability to perform activities of daily living, as compared to a prominent static neck collar (i.e., the HeadUp collar) developed for patients with DHS. This contribution provides important directions for future engineering development as well as biomechanical and clinical evaluations of the powered neck exoskeletons in patients with DHS.

## II. Methods

### A. Activity of Daily Living Tasks

We selected 8 tasks based on a literature review on the role of head-neck movements during activities of daily living [5], [21]. Sixteen activities were previously investigated that require a high ROM of the spine. Some of these activities require a higher ROM of the cervical spinal segment, while others need a greater ROM of the lumbar or thoracic spinal segment. We ranked the tasks by the contribution of the range of motion of the cervical spinal region (i.e., sum of the ROM in three anatomical planes). Overall, the top 8 tasks (Table I) were chosen where the head-neck contributes at least 50% of the total ROM of the three spinal segments. Additionally, we added a task “putting on and taking off a neck device” in this study, which was performed at the end of using each device. The time needed to don and doff the device was measured for this task (see section II-E).

### B. Devices

Two devices, i.e., the Utah Exo and the HeadUp collar, were used in this study. The Utah Exo allows up to 45° flexion, 15° extension, 20° left and right lateral bending, and 32° left and right axial rotation, based on previous bench results [20].

The Utah Exo is actuated by three servo motors (Dynamixel XM430-w350-r, ROBOTIS, Seuol, South Korea). The motors are all mounted on the shoulder pads to actuate the proximal revolute joints, thereby minimizing moving inertia caused by the motors during head motions. As a result, the robot felt light when moving the head [23]. The robot is also very back-drivable due to the robot’s kinematics structure. We have shown that it can be used to measure free head motions when the output torques of the servo motors are disabled [22]. In this study, we chose to use the robot in this mode to allow for the free motion of the head in healthy participants, constrained only to the mechanical structure of the robot.

The HeadUp collar (TalarMade Ltd, Chesterfield, UK) was chosen as the best-available, clinic-standard static neck collar, based on previous studies [9], [10]. The HeadUp collar comprises a soft piece of fabric in contact with the user’s neck and a series of thermoplastic beams that can be placed on different sides of the collar according to the patient’s needs and the guidelines provided in [24]. The beams came in two sets with two levels of flexibility, and each can be attached to the fabric in one of eight possible configurations. In this study, we chose to use the more flexible beams in a configuration that supported the head in all three directions (flexion and extension, axial rotation, and lateral bending).

Specifically, a trapezoid-shaped beam was attached between the chin and the chest to restrict the flexion of the head, two Z-shaped beams in conjunction with additional two narrow straight beams were attached between each side of the jaw and clavicle to restrict lateral bending of the head, and two wide straight beams were attached to the back of the neck to restrict the extension of the head.

In this setting, the maximum ROM allowed by the HeadUp collar when supporting the head from all sides can be measured. The HeadUp collar was available in two sizes for participants: medium and small. We chose the size of the HeadUp collar for each participant based on their neck circumference on the size guide table of the instruction manual [24]. As a result, all female participants received the small size while all male participants received the medium size in this study.

### C. Participants

12 healthy participants (6 male and 6 female; age: 26.1±3.5 years; height: 173.1±7.5 cm; neck circumference: 34.8±4.0 cm) without a known history of neck and head injuries were included in this study. None of the participants had experience using neck support devices before this study. This study was approved by the Institutional Review Board at the University of Utah (IRB #00145893). Written consent was obtained before any experimental procedure and data acquisition. Details pertaining to the study participants can be found in the table attached in the supplemental material.

### D. Measurement and Instrumentation

**Fig. 2.**
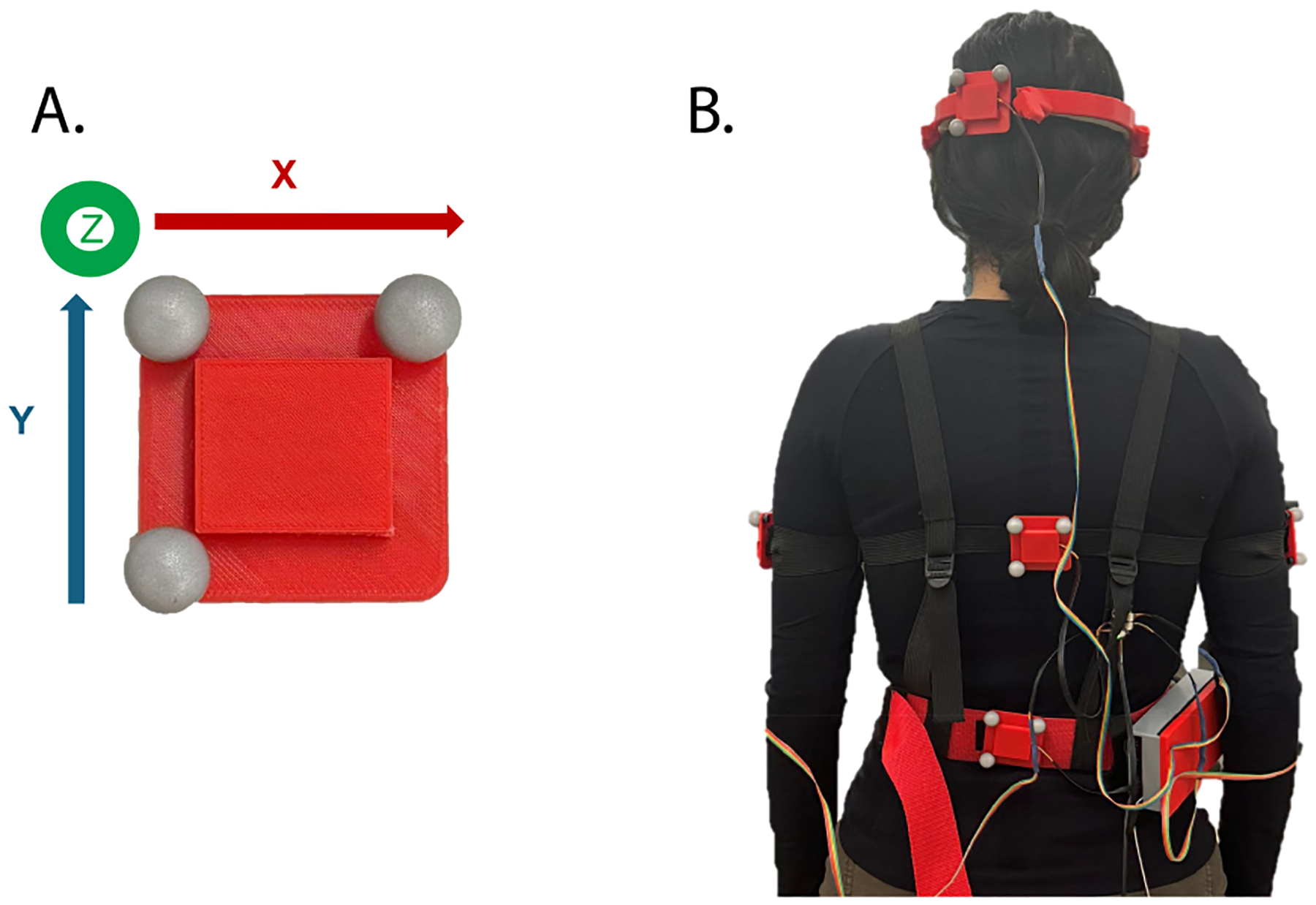
Experimental setup. A. Three infrared markers were placed on the corners of a plate to form a local coordinate system. The local coordinate system was formed by the x-axis pointing right (rotation axis for flexion-extension), the y-axis pointing up (rotation axis for axial rotation), and the z-axis pointing outward from the page (rotation axis for lateral bending). B. Three plates were placed on the head, upper thoracic, and lower lumbar regions through a headband and straps. Note that each marker plate also embeds a small and light inertial measurement unit (BNO055, Adafruit) which also recorded the rotations of these body segments. These data were collected for internal evaluations only and are not included in this paper.

A 12-camera motion capture system (Vero, Vicon, Oxford, UK) was used to measure the kinematics of three body segments (head, thoracic, and lumbar regions). The motion capture system was sampled at 100 Hz. An inertial ground frame was set after calibrating the cameras. The kinematics of the three body segments were measured with respect to the inertial frame using infrared markers. Three markers were mounted on a 3D-printed plate (Fig. 2A) to form the local coordinate system for each body segment.

For the head, the marker plate was placed on the robot headband, which was then attached to the forehead, consistent with the robot headband placement throughout the experiment. Custom-made elastic straps were used to attach the marker plates for the thoracic and lumbar areas (Fig. 2B).

### E. Procedure

Each device was properly secured on each participant. Each participant performed the 8 selected activities of daily living tasks (Table I) with five repetitions for each in three conditions: baseline (no neck support), wearing Utah Exo, and wearing a HeadUp collar. The baseline condition was performed first to provide a reference for the natural range of motion for the tasks. The order for wearing the Utah Exo and HeadUp collar was randomized following a balanced design: half of the participants used the Utah Exo first, while others used the HeadUp collar first. After using a device, responses to a questionnaire were collected (Questionnaire items can be found in Table III in section III-E). All participants had a practice trial for each task in each condition. The participants were asked to perform the tasks as naturally as possible at their preferred speeds and to keep the standing neutral pose for three seconds between repetitions /textcolorredto segment the repetitions. Note that the participants were not allowed to move their feet during the tasks. We also recorded the time needed to don and doff each device, where the participants were asked to put on and take off each device three times at the end of that device.

### F. Qualitative Assessment

We used a questionnaire with fifteen questions to gather participants’ perceptions of both neck support devices (details of questions are on Table III). This questionnaire was adapted from the survey used in [9]. The questionnaire consists of questions on how much the Utah Exo or HeadUp collar restricts their natural movement while performing each task. It also contains questions on the perceived comfort of each device for drinking, speaking, and breathing as well as the acceptability of the device weight. For each question, participants responded using a 5-point Likert scale (“strongly disagree”, “disagree”, “neutral”, “agree”, and “strongly agree”). Numerical values were assigned ranging from −2 for “strongly disagree”, to 0 for “neutral”, and to +2 for “strongly agree”.

### G. Data Processing and Statistical Analysis

As shown in Fig. 2, a local coordinate system was formed by the three infrared markers for each body segment (i.e., head, thoracic, and lumbar regions). Post processing, including labeling and gap filling of marker trajectories, was performed in software (Nexus, version 2.16.0). All movements are reported relative to the neutral position of the participant at the beginning of each trial, which was sitting upright with head facing forward for “backing up a car” and standing straight with head facing forward for all the other tasks. From the rotations of the bodies (head, thoracic, and lumbar) relative to the ground frame, we then calculated the relative rotations of the head to thoracic and the thoracic to lumbar. All rotations were then described using the axis-angle representation. Using the 3-second standing pose between repetitions, we segmented the kinematic trajectories for each repetition. We then computed the ROM of the head (relative to thoracic) and thoracic (relative to ground) using the maximum and minimum values of the trajectories in each anatomical plane (i.e., sagittal, coronal, and transverse planes).

The Shapiro-Wilk test was first used to examine the normality of the distributions of ROM of the head in each condition (without a device) for each task. Next, to find out head ROM on which anatomical plane is more important than others for each task, we compared the three components of the head ROM for each task in the baseline condition. Because the data were not normally distributed, we used the Kruskal-Wallis test. If there existed a head ROM component that is significantly higher than components on the other two planes in a task, we then selected this head rotation component as the primary head rotation for comparisons between conditions for the task. If there were two components of the head ROM that are similar to each other but significantly greater than the third one in a task, we then used both ROM components as the primary head rotations in the comparisons between conditions separately for the task.

Once the primary head ROM was determined by task, we compared this variable between conditions for each task using the Friedman test, followed by post-hoc Wilcox pairwise comparison test. This was to determine the effects of the condition on the primary head ROM for each task. Additionally, we compared the thoracic ROM in a similar fashion to determine the effect of conditions on the amount of compensatory movement of the rest of the body for each task. The effects of the order of the devices and sex were also analyzed using the Kruskal-Wallis test between conditions. Furthermore, the time needed to put on and take off a device were compared between the device conditions using a paired t-test. The users’ responses to each questionnaire item were compared between devices using the Wilcox pairwise comparison tests. All statistical significance level was set at *α* = 0.05.

## III. Results

### A. Primary Head Range of Motion

As summarized in Table II, three tasks (“looking for traffic”, “backing up a car”, “twisting with an object”) required predominantly axial rotation of the head, i.e., the head ROM in axial rotation was significantly greater than those in flexion-extension (*p* = 7.4 × 10^−7^, *p* = 7.4 × 10^−7^, *p* = 3.0 × 10^−6^, respectively) and lateral bending (*p* = 7.4 × 10^−7^, *p* = 7.4 × 10^−7^, *p* = 5.0 × 10^−5^, respectively). Meanwhile, three tasks (“picking up an object”, “reaching for a shelf”, “drinking water”) required much greater head flexion-extension than axial rotation (*p* = 1.4 × 10^−4^, *p* = 3.3 × 10^−5^, *p* = 6.6 × 10^−4^, respectively) and lateral bending (*p* = 7.4 × 10^−7^, *p* = 1.5 × 10^−6^, *p* = 5.0 × 10^−4^, respectively). In two tasks, “clearing table” and “simulating shower” required similarly large head ROM in axial rotation and flexion-extension (*p* = 0.41, *p* = 0.08). Both head ROM in flexion-extension and axial rotation were significantly greater than the head ROM in lateral bending in these two tasks (*p* = 3.7 × 10^−4^ and *p* = 6.6 × 10^−4^ for “clearing table”, and *p* = 7.4 × 10^−7^ and *p* = 1.4 × 10^−5^ for “simulating shower”). Notably, the head ROM in lateral bending was significantly less than the other two rotations in all tasks.

### B. Representative Kinematic Trajectory

**Fig. 3.**
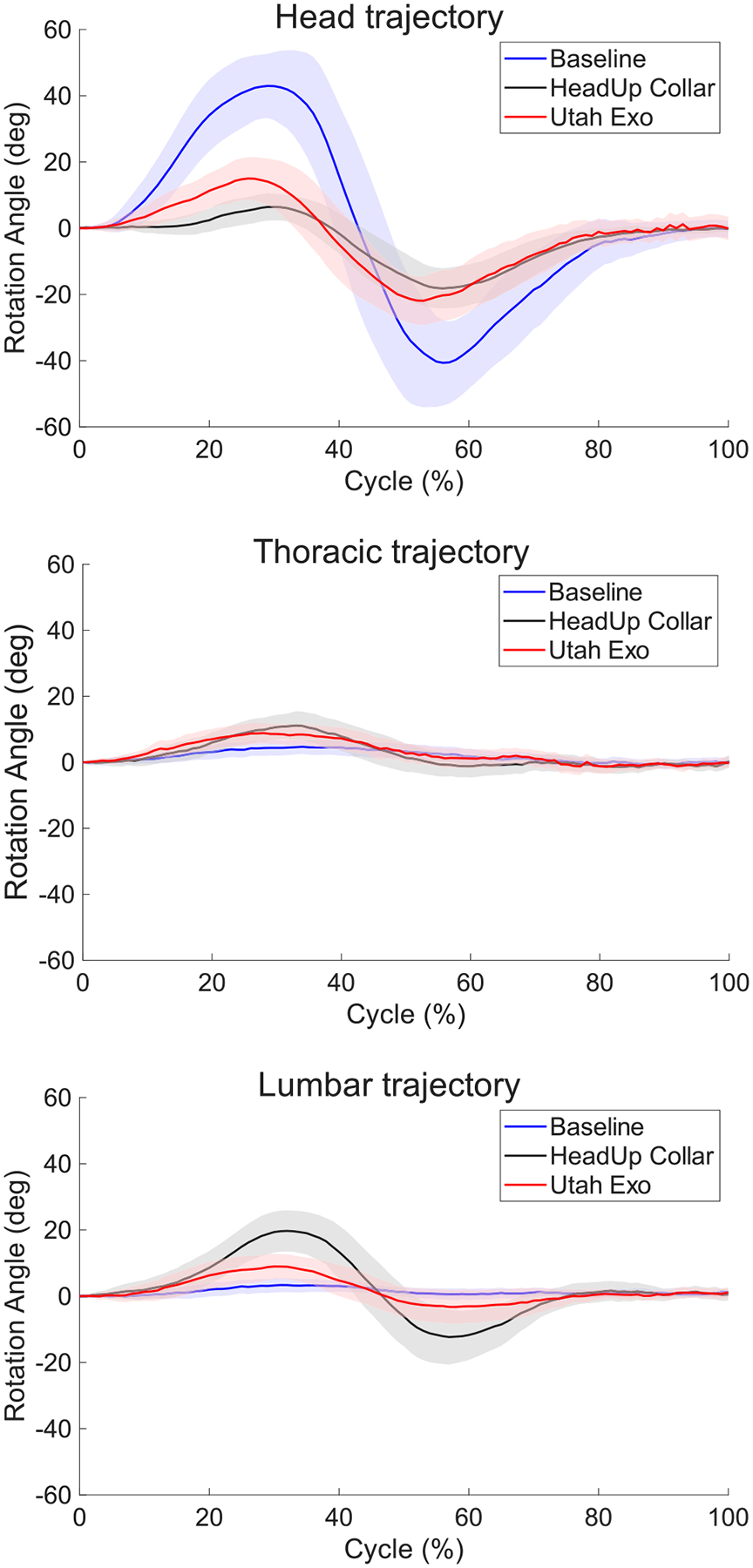
Head, thoracic and lumbar region axial rotation during “looking for traffic” task. This plot shows the mean trajectory of head, thoracic, and lumbar axial rotation angle in the looking for traffic task in three conditions. The blue line shows the baseline, the black line shows the HeadUp collar, and Utah Exo is in red. The band is the standard deviation among all participants and all their repetitions. Repetitions are normalized to a scale of 0–100. A video recording of this task in three conditions is available in the supplemental material.

The mean trajectory of the axial rotation of the head relative to thoracic, thoracic to lumbar, and lumbar to ground during the “looking for traffic” task in all three conditions is shown in Fig. 3 as the representative data. The mean head ROM needed to perform this task (baseline) was nearly 80°. This ROM is roughly 40° when Utah Exo was used, and 25° when the HeadUp collar was used. Movements in the thoracic region (relative to the lumbar segment) are similar between conditions, although the maximum angle was slightly higher when wearing either device than without a device. In contrast, movement in the lumbar region increased when a device was worn, and the change from the baseline was greater when the HeadUp collar was used. Although there was no obvious movement in the lumbar region during the baseline, the mean lumbar ROM reached approximately 20° and 40° in Utah Exo and HeadUp collar conditions, respectively.

### C. Group Results on Range of Motion

**Fig. 4.**
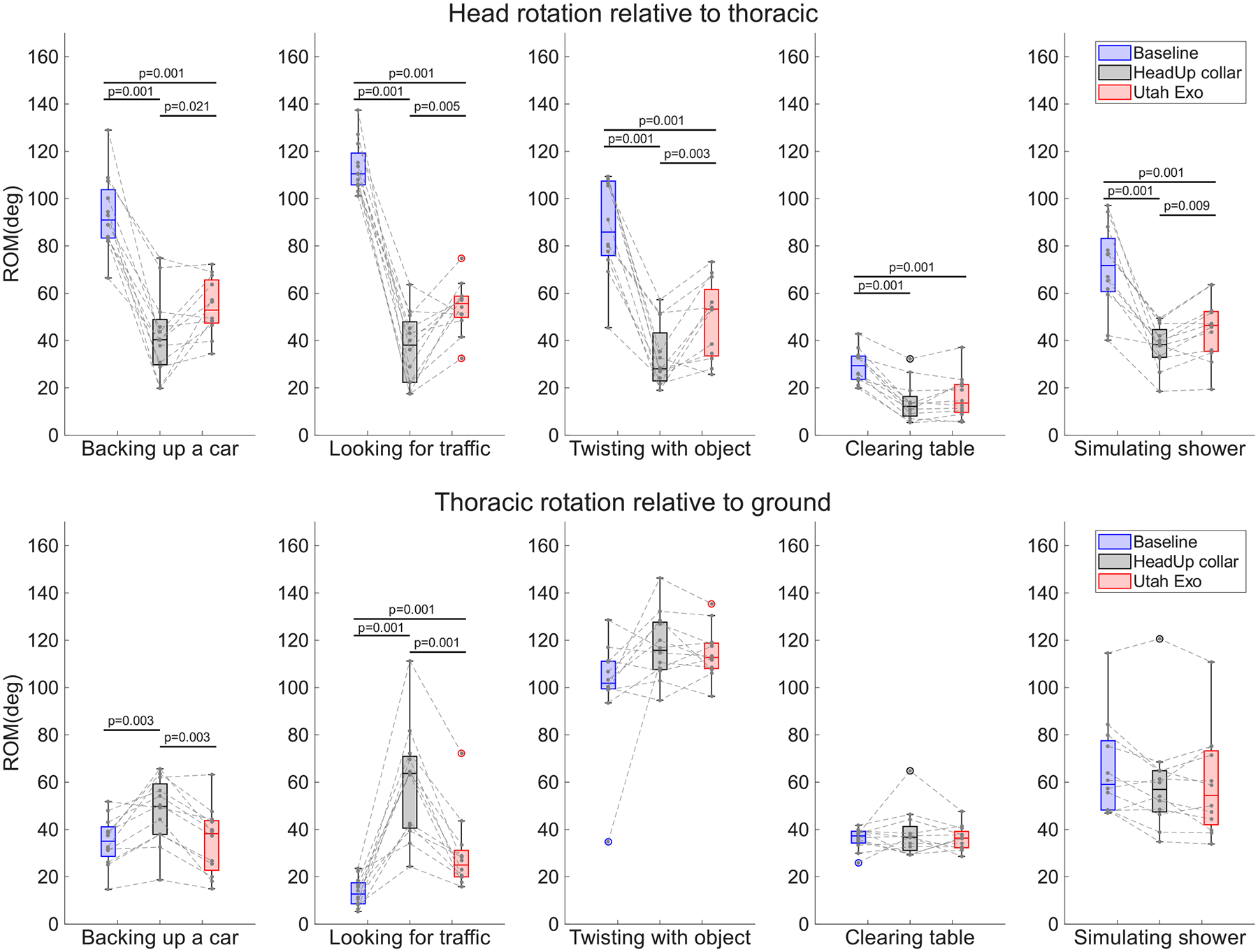
Axial Rotation ROM for head relative to thoracic and thoracic relative to ground. The top box plot shows the ROM of the head relative to the torso in the tasks with axial rotation as their dominant angle: “backing up a car”, “clearing table”, “looking for traffic”, “twisting with object”, and “simulating shower”. The bottom box plot shows the ROM of the thoracic relative to the ground in the same tasks. There are three boxes for each task, representing the condition, baseline is in blue, the HeadUp collar is in black, and the Utah Exo is in red. The black dots are the data points for each participant, and the black line between them connects the data points of one participant in three conditions.

**Fig. 5.**
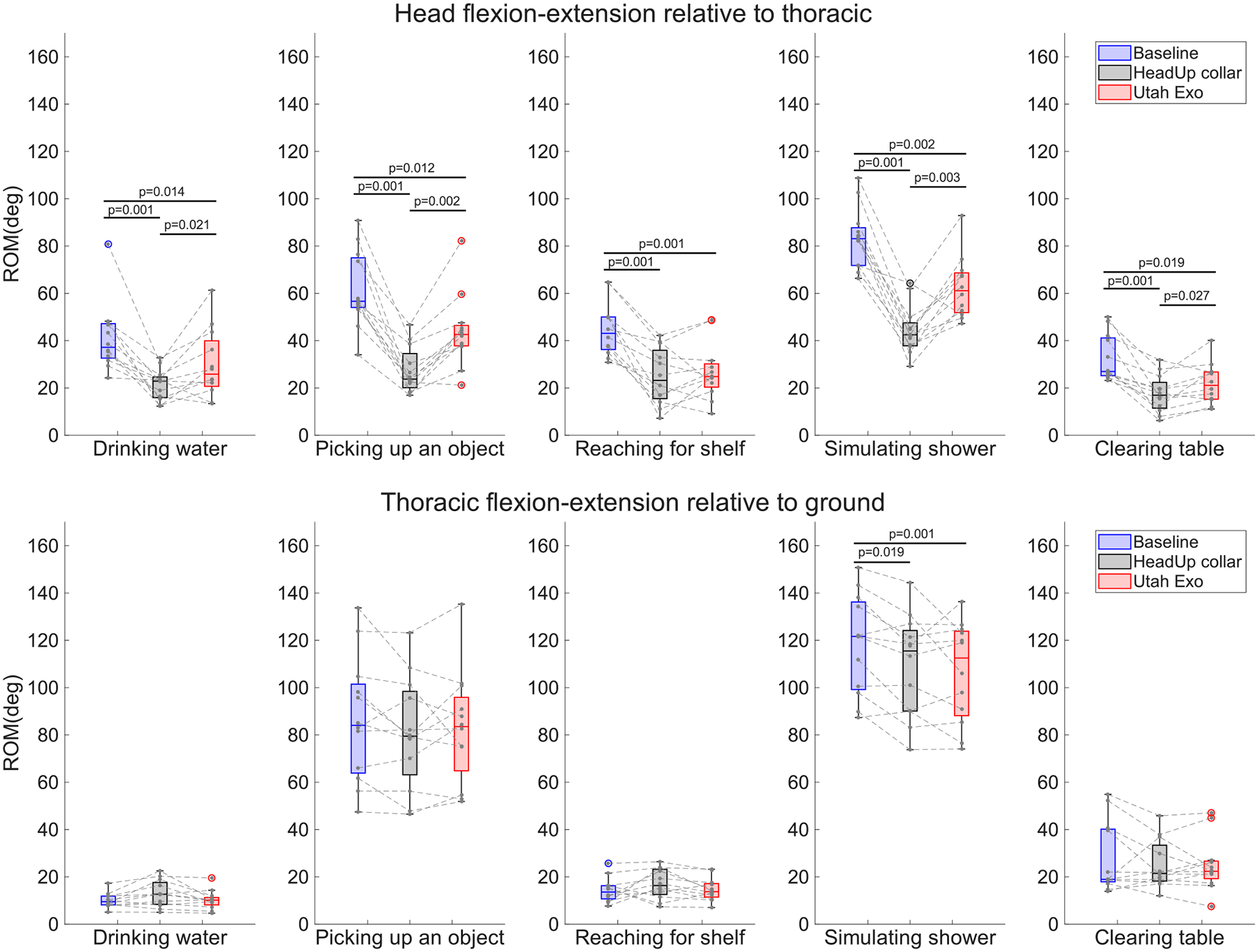
Flexion-extension ROM for head relative to thoracic and thoracic relative to ground. The top box plot shows the ROM of the head relative to the thoracic in the tasks with flexion-extension as their dominant angle: “drinking water”, “picking up an object”, “reaching for a shelf”, “simulating shower”, and clearing a table. The bottom box plot shows the ROM of the thoracic relative to the ground in the same tasks. There are three boxes for each task, representing the condition, baseline is in blue, the HeadUp collar is in black, and the Utah Exo is in red. The black dots are the data points for each participant, and the black line between them connects the data points of one participant in three conditions.

The group results were separated based on the primary motion of the tasks, as shown in Table II. Results of head ROM for tasks requiring more axial rotation are shown in Fig. 4 (top), while the results for tasks requiring greater flexion-extension motions are shown in Fig. 5 (top). Also shown are the compensatory movement, quantified using the thoracic ROM relative to the ground (Fig. 4 and Fig. 5 bottom). Note that the overall thoracic rotations included the contribution of the thoracic and lumbar segments.

During tasks requiring greater head axial rotation (Fig. 4 top), the ROM of the head during baseline is always greater than using either device (*p* = 0.001). When using the Utah Exo, the head ROM is almost always greater than using the HeadUp collar (*p* < 0.02), with the exception being the “clearing table” task (*p* = 0.2). In terms of the thoracic ROM (relative to ground), two tasks (“backing up a car” and “looking for traffic”) required greater compensatory movement of the torso when using the HeadUp collar, as compared to without a device or wearing the Utah Exo.

Similarly, for tasks involving more head flexion-extension, participants had the highest ROM without any devices (*p* < 0.05). The Utah Exo allowed for a greater ROM than the HeadUp collar (*p* < 0.05) in nearly all tasks, with the exception being the “reaching for shelf” task (*p* = 0.3). The compensatory movements do not show much difference between conditions in these tasks, with the exception of the “simulating shower”,task where the involvement of the torso was slightly higher (~ 5°) in baseline than using either device.

### D. Time to Don/Doff Devices

After completing tasks wearing a device, each participant put on and took off the device three times. Putting on the HeadUp collar (14.9 ± 4.1 seconds) took less time (*p* = 4 × 10^−6^) than putting on the Utah Exo (34.0 ± 8.0 seconds). Similarly, the time needed to remove the HeadUp collar (5.50 ± 1.63 seconds) was also less (*p* = 1 × 10^−4^) than removing the Utah Exo (7.8 ± 1.6 seconds).

### E. Questionnaire Results

Results from the questionnaires are summarized in Table III. Participants rated the devices negatively in four tasks (“looking for traffic”, “reaching for shelf”, “backing up a car”, “simulating shower”), based on their own natural movements for these tasks. In the other four tasks (“picking up an object”, “clearing table”, “drinking water”, and “twisting with an object”), participants rated the Utah Exo positively, while either being neutral or negatively towards the HeadUp collar, as compared to their natural behaviors. Participants rated the Utah Exo more favorably than the HeadUp collar (i.e., less restrictive) in four tasks: “picking up an object” (*p* = 3.2 × 10^−4^), “backing up a car” (*p* = 0.02), “clearing a table” (*p* = 1.3×10^−3^), and “drinking water” (*p* = 0.02). Additionally, the Utah Exo was perceived to be more comfortable (*p* = 0.01) and less restrictive for functions like speaking (*p* = 1.0×10^−3^), swallowing (*p* = 3.1 × 10^−4^), and breathing (*p* = 5.2 × 10^−3^). Compared against donning/doffing the HeadUp collar, participants regarded the Utah Exo to be slightly more difficult, although the difference was not significant (*p* = 0.76). Both devices have an acceptable weight for the participants.

### F. Influence of Order and Sex

The order of using both devices was randomized between participants. Six participants (3 male and 3 female) used the Utah Exo first, while the other six used the HeadUp collar first. There was no significant difference between these two groups (*p* = 0.5), indicating that the order had no influence on the results. On the other hand, male participants (N = 6) had greater head ROM than female participants (*p* = 0.001), suggesting an influence of sex on the results.

## IV. Discussion

We selected the activities of daily living for this study according to studies reported in the literature [5], [21]. Despite the ability of the head to rotate in three dimensions, most head movements for these daily tasks occurred in either flexion-extension or axial rotation. Lateral bending was not found to be important for healthy individuals to perform these tasks. When the mobility of the head is constrained, such as when using a head support device, compensatory movements of the torso, most likely in the lumbar segment due to its higher mobility than the thoracic segment, were observed.

One of the clinic-standard static neck collars [6], the HeadUp collar, was used in this study for comparison, which was considered an improved design from traditional neck collars [9]. While not completely restoring the head-neck motion needed for the selected daily tasks, our results did show a significant improvement in the ROM when using the Utah Exo, as compared to using the HeadUp collar. We showed that the Utah Exo improved the head ROM in nearly all tasks, and as a result, required much less compensatory body movements in some of the tasks (e.g., “backing up a car”).

Notably, our study involved healthy participants who could overcome the inherent stiffness imposed by the static neck collar. Additionally, the lower stiffness setting of the HeadUp collar (i.e., beams used around the neck were more compliant) was used for all participants. For patients with neck weakness who require a stronger support, like those with amyotrophic lateral sclerosis, it is likely that the head ROM would be much smaller when using the HeadUp collar.

Another piece of evidence supporting this conclusion is the much lower head ROM found in female participants when using the HeadUp collar, as compared to the male participants. Considering the identical stiffness setting in the HeadUp collar between sexes, this is likely contributed by the lower neck strength in the female participants [25].

Although putting on or taking off the Utah Exo took longer time than the HeadUp collar, the difference was only about 20 seconds.

A supporting video was attached to this manuscript to demonstrate the donning and doffing process of the two neck devices by a study participant.

Considering that a user would wear a device for multiple hours in a single use, this difference is not as significant from the user’s perspective, as shown from the survey responses. Additionally, the HeadUp collar requires additional treatment of the flexible components, according to the user’s manual [24]. These times, however, were not included in our experiment as we had prepared the HeadUp collar before the experiments. For patients with amyotrophic lateral sclerosis (neurodegenerative), it is very possible that the HeadUp collar would need multiple re-adjustments over the course of the disease progression, which may not be insignificant. In fact, as highlighted in [6], current static collars do not meet the evolving weakness in patients with neurodegenerative diseases, and thus a powered neck exoskeleton that use external actuators to provide support would potentially help overcome this important limitation for these users.

Another limitation of the HeadUp collar is that our participants felt restricted by the device when drinking, breathing, and speaking. They also reported that the overall comfort for the HeadUp collar is lower than for the Utah Exo. From the follow-up comments of the participants, this is likely because the HeadUp collar supports the head at the chin and around the neck, which creates pressures that limit these functions and overall comfort. As all existing neck collars employ a similar support strategy, this limitation is likely to apply to other static neck collars. This is especially problematic for patients with amyotrophic lateral sclerosis, because patients already face respiratory dysfunctions [6].

Our results showed that the Utah Exo required fewer compensatory torso movements in two tasks, “backing up a car” and “looking for traffic”, as compared to using the HeadUp collar. However, for all other tasks, there is no significant difference between using the Utah Exo and the HeadUp collar. Some tasks (e.g., “drinking water”, “picking up an object”) require much movement of the upper limbs, which may compensate for the lack of head ROM when using a neck device. For example, it was likely that a participant rotated the water bottle with the wrist instead of leaning the torso back when the head extension was limited by a neck device. Unfortunately, we did not record the hand motions in our study, which should be considered in future patient studies.

While allowing more head range of motion in most daily tasks using the Utah Exo, there is still a significant gap between what the exoskeleton allows and what is naturally required in most daily tasks. In the optimization of the Utah Exo, the ranges of motion in all three anatomical planes were weighed equally [20]. From our evaluation of head ROM for the selected daily tasks in this study, flexion-extension and axial rotation are more important than lateral bending. Therefore, future refinement of the neck exoskeleton should prioritize these two motions, which could result in a device that is more suited for activities of daily living.

Despite carefully securing the exoskeleton on the participants’ shoulders and head, there possibly existed small shifts of the device attachment on the user’s body. These device-on-body movements may have caused a slight overestimate of the head ROM for a couple of participants during two tasks requiring full-body movements (i.e., “simulating shower” and “picking up an object”). These suggest that potential improvement of the attachment and securing mechanism of the neck exoskeleton is needed for these movement tasks in the future.

The results from this work will help inform future study design for evaluations of the neck exoskeleton in patients with DHS. Data collected from the non-disabled participants will also provide the baseline healthy reference for comparisons in these future patient studies. Because the goal was to evaluate the mechanical factors of the neck exoskeleton for activities of daily living, we used the “transparent” mode of the neck exoskeleton to obtain the natural head-neck and torso movements in healthy individuals wearing this device. Although multiple viable controllers are available to assist with the head-neck movement [11], [16], [26], we did not use them in this study involving healthy participants. This is because, without proper personalization, the controller deficiencies will likely influence our kinematics results for the head-neck and torso. However, future investigations should focus on personalizing controllers and evaluating the neck exoskeleton in the powered modes for activities of daily living in patients with DHS.

We used a custom-made questionnaire in this study to gather subjective responses from the healthy participants. Each question was asked once, corresponding to each task, on a 5-point Likert scale. As a result, responses from participants were often in agreement due to the low resolution of the Likert scale. This is a limitation, because we cannot establish a reasonable correlation between the subjective response and the objective ROM measurement. A higher resolution Likert scale with possibly repeated measures is therefore necessary for such an analysis, which would be valuable to understand the biomechanical factors contributing to the user’s experience.

There exist limitations pertaining to the evaluation of the donning process of the two devices in the current study. Because healthy participants have the strength to hold their head up, they can put on and fit the HeadUp collar themselves. As its user’s manual [24] suggests, however, it may require a caregiver to help put on the HeadUp collar for a patient with DHS. By contrast, a patient with DHS may be able to self-don the neck exoskeleton by first attaching the headband and securing the shoulder straps (as shown in the supporting video), followed by switching to the powered mode to lift the head back up to the upright position via a controller. Unfortunately, because we only evaluated the donning and doffing process in healthy participants, we were not able to demonstrate this potential feature of the powered neck exoskeleton. We will include such evaluations with patients with DHS in the future.

In this study, we only collected the overall acceptance information from the participants due to the short duration of the study. Therefore, potential changes in user’s perception of, for example, the device weight across different usage durations were unknown. In future evaluations with patients with DHS, the questionnaire items will be further refined to include evaluations at different usage durations (e.g., initial wearing, 1 hour after wearing, half a day after wearing) to gain a more comprehensive understanding of how the user’s acceptance changes over time. Moreover, although the the impact of order and sex were considered, other potential influencing factors (e.g., baseline neck muscle strength) should be added in subsequent studies to better interpret the experimental data. Additionally, the present evaluation was confined within a laboratory motion capture space. We therefore were not able to assess other important daily tasks that are not stationary, such as navigating a hallway. Future studies should consider using mobile sensing, such as mobile cameras [27] or inertial measurement units, to evaluate this exoskeleton outside of the laboratory.

## V. Conclusion

This paper presented a study to evaluate the ROM during activities of daily living while wearing different neck supporting devices. Our results showed that the Utah Exo, a robotic neck exoskeleton, allows more head ROM for important daily activities, as compared to a clinic-standard static neck collar (HeadUp collar). The Utah Exo was also perceived to be more comfortable to wear for these tasks than the HeadUp collar. These results provided important justification of using powered neck exoskeletons to enhance daily functions. Future directions will include further improving design of the Utah Exo to allow higher ROM for activities of daily living and quantify effects of the Utah Exo (while being actively controlled) on the ability to perform activities of daily living in patients with DHS resulting from neck muscle weakness.

## Supplementary Material

supp2-3647266

supp1-3647266

This article has supplementary downloadable material available at https://doi.org/10.1109/TNSRE.2025.3647266, provided by the authors.

## Figures and Tables

**Fig. 1.**
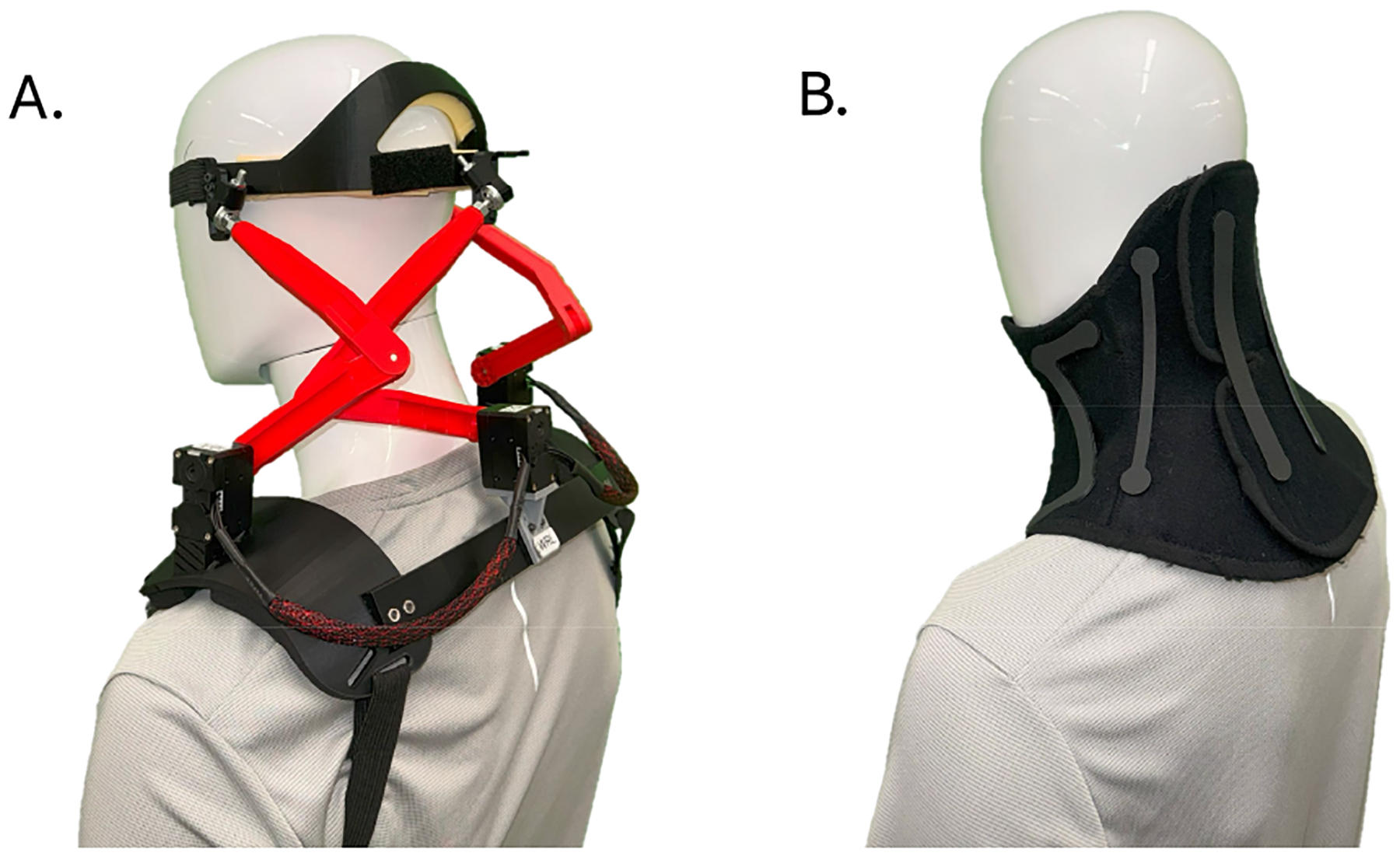
Devices used in the study. A. Utah exo. B. HeadUp collar.

**TABLE I:** Selected Activities of Daily Living

Order	Task	Description
1	Looking for traffic	Participant stands with a slight forward bend at the waist and turns head to left, then right as if looking for traffic before crossing the street.
2	Picking up an object	From an upright position, the participant picks up a towel off the floor. Then they return to an upright position and drop the towel on the floor.
3	Reaching for shelf	Participant stands and tries to reach a power outlet hanging from the ceiling 5 ft in front of the participant with their dominant hand.
4	Backing up a car	Participant sits on a stool with feet on the floor, rests right arm on the back of a chair placed next to him/her, simulates holding the steering wheel in the left hand, and looks over right shoulder as if backing up the car.
5	Clearing table	Participant stands against and reaches across a roughly waist-high table. Participant simulates grabbing a full 20-ounce water bottle, bringing it back across the table, and resuming a standing position.
6	Drinking water	Participant stands and is given a half-poured 20-ounce water bottle. The participant was instructed to take a small drink from the bottle.
7	Simulating shower	Participant stands and tries to reach all their joints from the opposite side of their dominant hand with their dominant hand, then switch the hand and do the same.
8	Twisting with an object	Participant stands between 2 roughly waist-high chairs. The participant keeps their feet on the floor and rotates to one side to pick up a 5-pound weight placed on a chair, then rotates to the other side, places the weight on the opposite chair, and places it back on the first chair.
9	Putting on and taking off the device	Participant stands facing the device sitting on the table, reaches for the device, puts the device on with no help, takes it off, and leaves it on the table in the same position as the first.

**TABLE II:** Primary Head Rotation for Each Task

Task	Primary Head Rotation
Looking for traffic	Axial rotation
Picking up object	Flexion-extension
Reaching for shelf	Flexion-extension
Backing up car	Axial rotation
Clearing table	Axial rotation, Flexion-extension
Drinking water	Flexion-extension
Simulating shower	Axial rotation, Flexion-extension
Twisting with object	Axial rotation

**TABLE III:** Questionnaire Questions and Summary of Results

Order	Question	Utah Exo mean rating (Mean and SD)	HeadUp collar mean rating (Mean and SD)	Wilcoxon signed-rank test
1	I feel wearing HeadUp Collar/Utah Exo does not interfere with my natural standing posture.	1.42±0.90	0.33±1.61	*p* = 0.14
2	I feel HeadUp Collar/Utah Exo does not restrict my natural movement while performing the “looking for traffic” task.	−0.75±1.0	−1.3±0.65	*p* = 0.13
3	I feel HeadUp Collar/Utah Exo does not restrict my natural movement while performing the “picking up an object” task.	1.0±0.43	−0.33±0.78	*p* = 3.2 × 10^−4^*
4	I feel HeadUp Collar/Utah Exo does not restrict my natural movement while performing the “reaching for a shelf’ task.	−1.1±1.2	−0.75±1.1	*p* = 0.37
5	I feel HeadUp Collar/Utah Exo does not restrict my natural movement while performing the “backing up a car” task.	−0.58±1.0	−1.5±0.67	*p* = 0.02*
6	I feel HeadUp Collar/Utah Exo does not restrict my natural movement while performing the “clearing table” task.	1.5±0.52	0.50±0.67	*p* = 1.3 × 10^−3^*
7	I feel HeadUp Collar/Utah Exo does not restrict my natural movement while performing the “drinking water” task.	1.0±1.3	−0.25±1.1	*p* = 0.01*
8	I feel HeadUp Collar/Utah Exo does not restrict my natural movement while performing the “simulating shower” task.	−0.75±1.2	−0.25±0.75	*p* = 0.20
9	I feel HeadUp Collar/Utah Exo does not restrict my natural movement while performing the “twisting with an object” task.	0.75±0.97	0.0±1.0	*p* = 0.08
10	I feel HeadUp Collar/Utah Exo does not restrict my ability to swallow.	1.1±1.0	−0.36±1.0	*p* = 5.2 × 10^−3^*
11	I feel HeadUp Collar/Utah Exo does not restrict my ability to breathe.	1.8±0.39	0.50±1.0	*p* = 3.1 × 10^−4^*
12	I feel HeadUp Collar/Utah Exo does not restrict my ability to speak.	1.9±0.29	0.33±1.2	*p* = 1.0 × 10^−3^*
13	I feel HeadUp Collar/Utah Exo is comfortable to wear.	0.083±0.79	−1.1±1.2	*p* = 0.01*
14	I feel HeadUp Collar/Utah Exo is easy to put on.	−0.17±1.1	0.000±1.4	*p* = 0.76
15	I feel HeadUp Collar/Utah Exo has an acceptable weight.	0.67±0.89	0.50±0.90	*p* = 0.58
