# Supplementary material for: Evaluating Range of Motion of Two Prominent Neck Support Devices for Daily Activities
Source: IEEE Trans Neural Syst Rehabil Eng. Author manuscript; Available in PMC 2026 Feb 9. (PMC12884773; doi:10.1109/TNSRE.2025.3647266)
Supplement: supp2-3647266 [file NIHMS2135939-supplement-supp2-3647266.pdf]

Supplemental Table: Characteristics of the study participants

Participant	Sex	Age (years)	Height (cm)	Neck circumference (cm)	First device used
P01	Male	26	170	36	HeadUp collar
P02	Male	21	185	37	Utah Exo
P03	Female	29	165	31	HeadUp collar
P04	Female	22	170	33	Utah Exo
P05	Male	28	175	37	HeadUp collar
P06	Female	26	170	33	HeadUp collar
P07	Male	30	175	42	Utah Exo
P08	Male	23	177	40	HeadUp collar
P09	Male	33	187	37	Utah Exo
P10	Female	25	167	30	Utah Exo
P11	Female	24	167	31	HeadUp collar
P12	Female	26	165	31	Utah Exo
